# Sestrin2 as a Protective Shield against Cardiovascular Disease

**DOI:** 10.3390/ijms24054880

**Published:** 2023-03-02

**Authors:** Muhammad Ammar Zahid, Shahenda Salaheldin Abdelsalam, Hicham Raïq, Aijaz Parray, Hesham Mohamed Korashy, Asad Zeidan, Mohamed A. Elrayess, Abdelali Agouni

**Affiliations:** 1 Department of Pharmaceutical Sciences, College of Pharmacy, QU Health, Qatar University, Doha P.O. Box 2713, Qatar; 2 Department of Social Sciences, College of Arts and Sciences, Qatar University, Doha P.O. Box 2713, Qatar; 3 The Neuroscience Institute, Academic Health System, Hamad Medical Corporation (HMC), Doha P.O. Box 3050, Qatar; 4 Department of Basic Medical Science, College of Medicine, QU health, Qatar University, Doha P.O. Box 2713, Qatar; 5 Biomedical Research Center (BRC), Qatar University, Doha P.O. Box 2713, Qatar; 6 Office of Vice President for Research and Graduate Studies, Qatar University, Doha P.O. Box 2713, Qatar

**Keywords:** Sestrin2, cardiovascular disease, cellular stress, cardioprotective, oxidative stress, antioxidant

## Abstract

A timely and adequate response to stress is inherently present in each cell and is important for maintaining the proper functioning of the cell in changing intracellular and extracellular environments. Disruptions in the functioning or coordination of defense mechanisms against cellular stress can reduce the tolerance of cells to stress and lead to the development of various pathologies. Aging also reduces the effectiveness of these defense mechanisms and results in the accumulation of cellular lesions leading to senescence or death of the cells. Endothelial cells and cardiomyocytes are particularly exposed to changing environments. Pathologies related to metabolism and dynamics of caloric intake, hemodynamics, and oxygenation, such as diabetes, hypertension, and atherosclerosis, can overwhelm endothelial cells and cardiomyocytes with cellular stress to produce cardiovascular disease. The ability to cope with stress depends on the expression of endogenous stress-inducible molecules. Sestrin2 (SESN2) is an evolutionary conserved stress-inducible cytoprotective protein whose expression is increased in response to and defend against different types of cellular stress. SESN2 fights back the stress by increasing the supply of antioxidants, temporarily holding the stressful anabolic reactions, and increasing autophagy while maintaining the growth factor and insulin signaling. If the stress and the damage are beyond repair, SESN2 can serve as a safety valve to signal apoptosis. The expression of SESN2 decreases with age and its levels are associated with cardiovascular disease and many age-related pathologies. Maintaining sufficient levels or activity of SESN2 can in principle prevent the cardiovascular system from aging and disease.

## 1. Introduction

An adequate and timely response to stress is a necessity for all cells to remain viable and functioning. Cellular mechanisms that are activated in response to stress depend on the type of stress, although common effects are often observed. For example, DNA damage, ischemia/hypoxia, and endoplasmic reticulum (ER) stress all lead to increased oxidative stress with the accumulation of reactive oxygen species (ROS). Mitochondrial dysfunction due to structural lesions plays an important role in the accumulation of ROS as electrons from the oxidative phosphorylation chain leak into the cytoplasm and generate highly reactive free radicals which, in turn, can react with other macromolecules and cellular structures. The conservation of energy at this stage is of prime importance to keep driving the essential cellular machinery. A shift in the metabolism from adenosine triphosphate (ATP) consuming anabolic reactions to ATP-producing catabolic reactions takes place. The shift of metabolism from anabolism to energy-producing catabolism serves two purposes; the production of energy to keep diving the cellular machinery in the stressed state and the degradation of macromolecules and organelles that are damaged. When the stress is beyond control, programmed cell death or apoptosis is another consequence that removes the severely damaged cells without collateral damage to the surrounding tissues. If necrosis occurs instead of apoptosis, an inflammatory response could initiate contributing, thus, to further tissue damage. Death and removal of damaged cells also prevent the malignant transformation of these cells. The immune system also plays an important role in programmed cell death. The disruption in functioning and coordination of the stress response has pathological consequences, such as cardiovascular disease (CVD), cancer, diabetes, and neurodegenerative diseases. Aging comes with a natural decline of cellular stress responses which contributes to the accumulation of cellular lesions, pathologies, and death. Understanding the molecular pathways involved in the stress response and the role of different proteins can lead to new drug targets to augment the stress response, slow down the onset of age-related pathologies, or enhance the degradation of damaged cells to prevent cancerous transformation. Many proteins which play a role in stress are multi-functional and Sestrin2 (SESN2) has recently attracted interest as one of those important proteins.

Genomic instability, disruptions in protein balance, changes in how cells respond to nutrients, mitochondrial dysfunction, cellular senescence, depletion of stem cells, and altered communication between cells are all hallmarks of aging and contributes to aging in one way or another [1]. Activation of AMP-activated protein kinase (AMPK), inhibition of the mammalian target of rapamycin complex 1 (mTORC1) activation, and activation of the autophagic signaling have been shown to increase the life and health span of model organisms [2,3,4,5]. SESN2 has been shown to activate AMPK, inhibit mTORC1, activate autophagy, and regulate energy and metabolic homeostasis [6,7,8]. Thus it seems plausible that SESN2 also contributes to suppression of age-associated diseases. In fact, increased levels of DrosophilaSestrin (dSesn) were observed in chronic TOR activation due to accumulation of ROS [9]. Loss of *dSesn* resulted in age-related pathologies, such as muscle degeneration, cardiac malfunction, triglyceride accumulation, and mitochondrial dysfunction, which were prevented by activating AMPK or inhibiting TOR. dSesn acted as a negative feedback regulator of TOR, integrating metabolic and stress inputs to prevent pathologies caused by chronic TOR activation [9]. Sestrins also protect the cells from physiological day-to-day stress resulted from metabolic activities, such as oxidative respiration and DNA replication, as *dSesn* mutants showed an accelerated aging phenotype even in the absence of any environmental stress. The deregulation in the metabolic homeostasis as evident by the increased lipids and sugar levels in the blood were also observed in *dSesn* null *Drosophila* and *SESN2*-knockout mice [9,10]. SESN2 levels in aging cardiac muscles have been shown to decrease with age and contribute to the decreased stress tolerance [11,12,13]. Taken together, reduced cardiac stress tolerance and metabolic deregulation contribute greatly to the development of CVD.

CVD is a leading cause of death worldwide [14]. Stress on endothelial cells and cardiomyocytes, which make up the bulk of the cardiac tissue and vessels, due to changing metabolism, hemodynamics, or oxygenation status is at the core of these diseases [15,16,17]. The ability to cope with stress depends on the expression of endogenous stress-inducible molecules. One of such stress-inducible protein-coding genes, *SESN2*, initially known as hypoxia-induced genes 95 (*HI95*), was discovered in an attempt to identify genes that control the viability and fate of cells during prolonged hypoxia [18]. SESN2 belongs to the family of highly conserved proteins inducible by many different types of stress conditions, including oxidative stress, genotoxic stress, ER stress, hypoxia, and energetic and metabolic stress. Once induced, SESN2 fights back through an intrinsic antioxidant enzymatic activity [19], the activation of AMPK [6], the inhibition of mTORC1 [20,21], the activation of nuclear factor erythroid 2-related factor 2 (NRF2) [22], the activation of AKT [23] and autophagy [24]. Genetic deletion of *SESN2* has been shown to worsen age-related pathologies and cardiac function in a variety of model organisms [9,10,25,26]. In cardiomyocytes, *SESN2* deletion worsens oxidative and ER stress and contributes to cardiac dysfunction [25,26]. SESN2 also prevents intolerance to ischemia/reperfusion injury which occurs in aging [11]. A decline in SESN2 levels with age is well known but a recent association study of *SESN2* polymorphism with the risk of congenital heart defects has shown a genetic predisposition to reduced levels of SESN2 which contributes to low cardiomyocyte viability under hypoxic stress [27]. Further research is needed in this regard to ascertain how genetic or epigenetic changes can alter endogenous SESN2 levels and if the increase in endogenous SESN2 levels or activity can be a therapeutic option to prevent the cardiovascular system from stressful conditions. In this article, we have reviewed the upstream regulators of SESN2 in response to different types of cellular stress and the downstream pathways affected by SESN2. The current evidence of the protective role of SESN2 in CVD and the possibility of SESN2 as a drug target is also presented.

## 2. *SESN2* Gene and Protein Structure

The family of Sestrins consists of three homologous members: SESN1, SESN2, and SESN3. All three Sestrins are encoded by genes located on different chromosomes. The *SESN1* gene located on chromosome 6q21 also referred to as PA26, is a transcriptional target for the tumor suppressor P53 and is induced in response to genotoxic stress, such as UV radiations [28]. The *SESN2* gene located on chromosome 1p35.3 was discovered in an attempt to identify genes induced by prolonged hypoxia and is also referred to as *HI95* [18]. Database mining and bioinformatic analysis revealed another PA26-related gene which was named after Sestrins as *SESN3* and is present at locus 11p21 [29].

**Figure 1:**
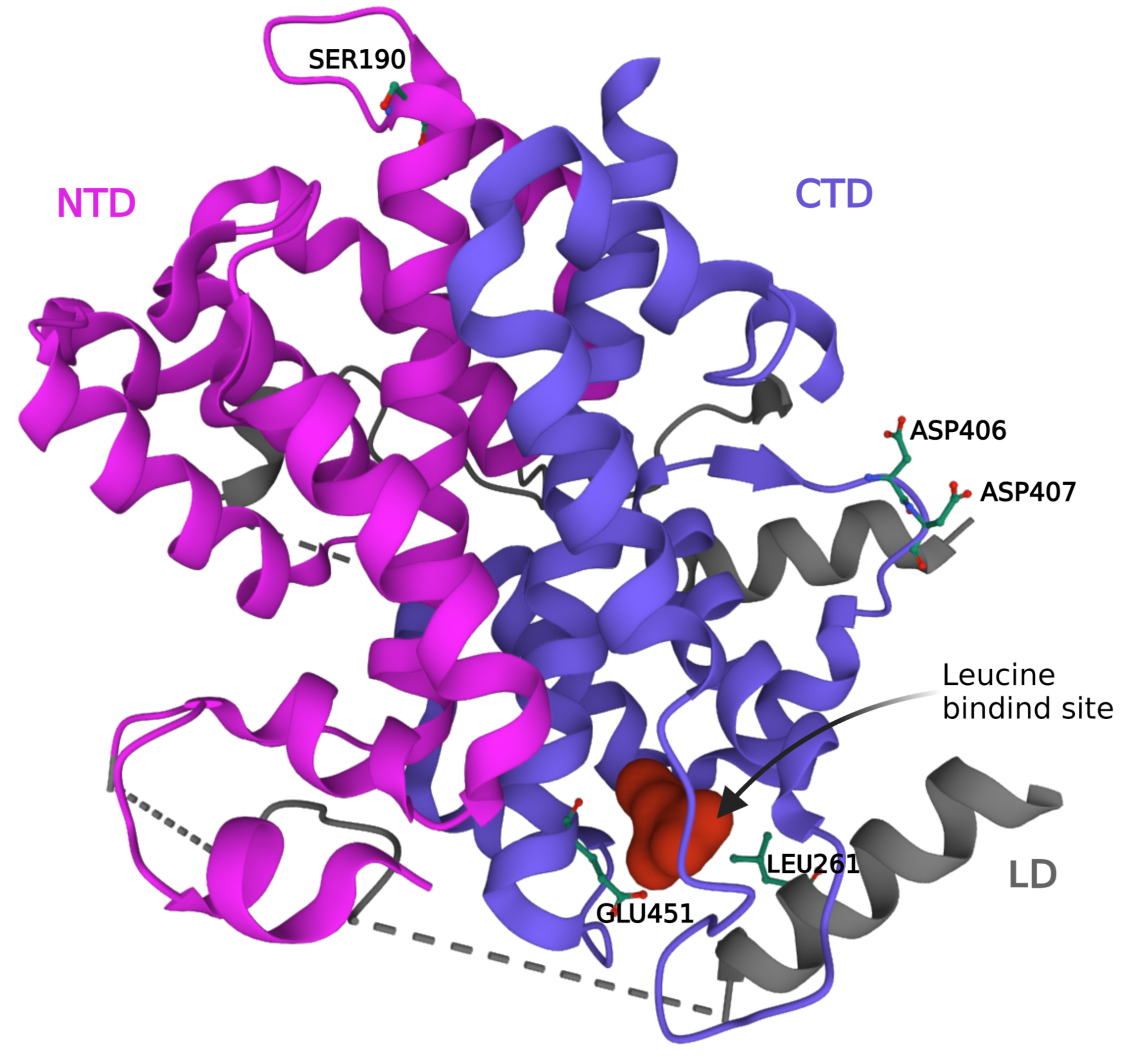
Leucine bound structure of SESN2 (PDB 5DJ4). The protein consists of an NTD and CTD joined together by a small LD. The leucine binding site is present in the CTD. The residues important for leucine binding (GLU451 and LEU261) and the interaction of GATOR2 (ASP406-407 and SER190) with SESN2 are labeled. CTD: C-terminal domain; LD: Linker domain; NTD: N-terminal domain.

The *SESN2* gene encodes for a single monomeric protein of 480 amino acids and a mass of 55 kDa. SESN2 is a globular protein comprising only α-helices and no β-sheets (Figure 1). The X-ray crystallographic structures (PDB ID: 5DJ4) contains an N-terminal domain (NTD) comprising of residues 66–220, a C-terminal domain (CTD) comprising of residues 339–480, connected by a hinge-like linker domain (LD) comprising of residues 221–338 [30]. NTD, LD, and CTD correspond well to SESN-A, SESN-B, and SESN-C identified previously by the primary sequence and phylogenetic analysis. NTD and STD have a low primary sequence similarity but superimpose well on one another and resemble AphD monomer. The leucine binding pocket is present in the CTD at the intersection of three helices and an LD helix packs against the pocket. Amino acid residues GLU451 in the CTD and LEU261 in the LD are critical for the binding of leucine to SESN2 [30]. The amino acid residues important for the interaction of GATOR2 with SESN2 are SER190 in the NTD and ASP406 and ASP407 in the CTD [31]. The proximity of ASP406 and ASP407 to the leucine binding pocket can explain the changes in the interaction of SESN2 with GATOR2 in the presence of leucine. A detailed representation of the structural details for the interaction of SESN2 with other proteins, such as AMPK and LKB1, in coordinating the stress response and effect of leucine binding on these interactions is still elusive. This structural information will help to discover and design small molecules capable of stabilizing these interactions to augment the activity of SESN2.

## 3. Upstream Regulators of SESN2 Expression in Response to Different Types of Cellular Stress

SESN2 is a stress-inducible protein and is expressed in response to different types of stresses. In return, the increase in expression of SESN2 tends to normalize the stress and fight for the survival of the cell, directly by using its antioxidant enzymatic activity and indirectly by regulating several signaling pathways. In this section, the regulation of SESN2 expression by different stresses is reviewed. It is worth mentioning that although for the sake of understanding, regulation of SESN2 expression in response to the different types of stress has been discussed separately, this regulation is interlinked, not so simple, and straightforward as one type of stress may progress into another type very often and rapidly. For example, an initial episode of hypoxia can progress into energetic stress. Oxidative stress can alter nucleic acids and proteins leading to genotoxic and ER stress. Thus, these regulatory mechanisms work in close coordination with one another and not simply in isolation.

### 3.1. Hypoxia

**Figure 2:**
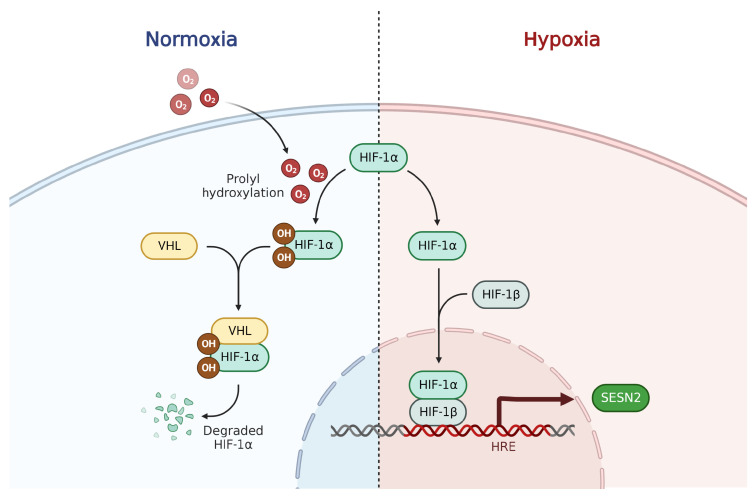
The regulation of SESN2 in response to hypoxic stress. During normoxia, the cellular levels of HIF-1α remain low due to VHL-mediated proteasomal degradation facilitated by the hydroxylation of the proline residues. Hypoxia prevents the degradation of HIF-1α and in association with HIF-1β, the complex translocates to the nucleus to regulate the expression of genes under its transcriptional control. HRE: Hypoxia Response Element; VHL: Von Hippel–Lindau tumor suppressor.

Loss of blood supply to a tissue leads to the decreased partial pressure of oxygen in cells and can lead to pathological tissue damage if not corrected. It is well-known that hypoxia triggers a defense mechanism by signaling through the hypoxia-inducible factor 1-alpha (HIF-1α) [32]. HIFs are transcription factors that are constantly degraded in normoxia but are stabilized by hypoxia and increase the expression of different stress-related genes including *SESN2* [33]. SESN2 was originally identified as a protein that is inducible by hypoxia independent of the involvement of p53 [18]. Budanov et al. used deferoxamine to model prolonged hypoxia in cells, which is a stabilizer for HIF-1α. Many other studies have shown that SESN2 induction in response to hypoxia is HIF-1α-dependent [34,35]. A schematic representation of the induction of SESN2 by HIF-1α stabilization in hypoxic conditions is shown in Figure 2.

### 3.2. Genotoxic Stress

**Figure 3:**
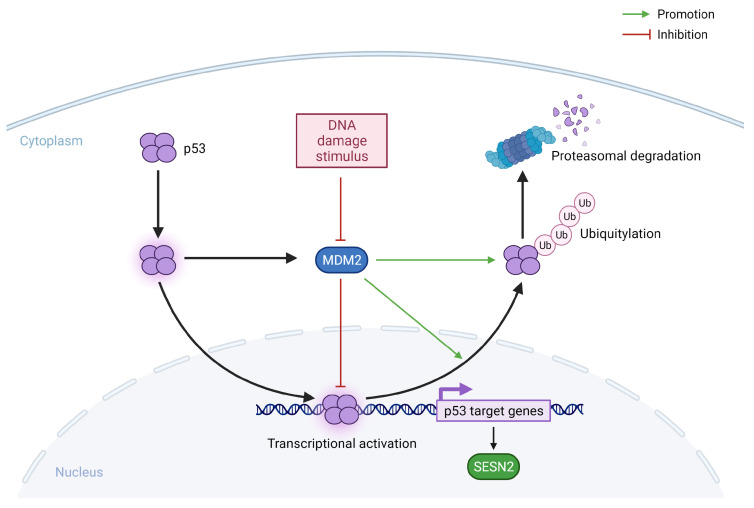
The regulation of SESN2 in response to genotoxic stress. The levels of p53 are kept low via the process of ubiquitination by MDM2 and subsequent proteasomal degradation. Post-translational modifications of MDM2 and p53 in response to genotoxic stress disrupt the interaction between MDM2 and p53, increasing the cytosolic concentration of p53 and its nuclear translocation, and eventually the transactivation of the target genes. MDM2: Mouse Double Minute 2 homolog; Ub: Ubiquitin.

P53 is one of the important molecules which decide the fate of the cell in response to damage in the DNA [36]. In resting cells without genotoxic stress, p53 levels remain low due to continuous ubiquitination and proteasomal degradation by mouse double minute 2 homolog (MDM2) ubiquitin ligase [37]. The activation of p53 in response to DNA damage is a complex process and involves stabilization and activation by post-translational modifications of p53 and MDM2 [38]. Once stabilized and activated, p53 moves to the nucleus and causes the transactivation of genes involved in the arrest of the cell cycle, apoptosis, and senescence including *SESN2* (Figure 3). The arrest of the cell cycle gives enough time for the DNA damage to repair and to prevent the damage to propagate in the progeny cells. However, if the damage is beyond repair, p53 can signal apoptosis of the cell. In the initial experiments by Budanov et al., *SESN2* was identified as a p53-target gene and it was shown that the induction of SESN2 in response to DNA-damaging treatments, such as gamma and UV radiations or doxorubicin, is dependent on a functional p53. Cell lines containing a mutated form of p53 do not show an increase in SESN2 expression in response to these stresses. Further experiments by the same group confirmed that p53 is what connects genotoxic stress to increased expression of SESN2 and mTORC1 inhibition because the silencing of *SESN2* attenuates the inhibitory effect of p53 over mTORC1 [20]. Since then, many groups have provided additional evidence of the regulation of SESN2 expression by p53 in response to different types of stresses causing actual or potential damage to DNA [24,39].

### 3.3. Endoplasmic Reticulum Stress

**Figure 4:**
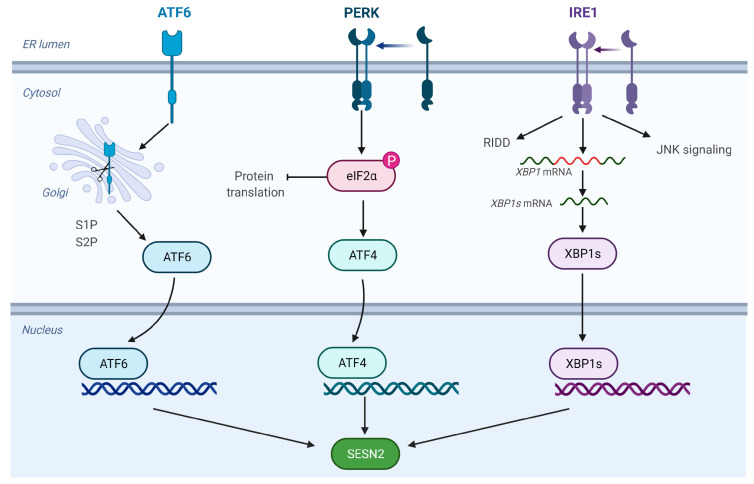
The regulation of SESN2 in response to ER stress. The UPR sensors—PERK, IRE-1α, and ATF-6—remain inactive by binding to BIP under non-stressed conditions. Accumulation of misfolded proteins during ER stress results in the dissociation of BIP induces dimerization of PERK and IRE-1α, as well as transport of ATF-6 to the Golgi apparatus. IRE-1α endoribonuclease activity generates an important transcription factor XBP-1s, which enters the nucleus to regulate the expression of *SESN2*. The severe and chronic ER stress response causes the activation of RIDD and JNK. PERK induces the phosphorylation of the α subunit of translation initiation factor 2 (eIF-2α), which blocks the translation initiation. PERK activation paradoxically promotes the transcription initiation of the gene encoding ATF-4 which induces *SESN2*. ATF-6 moves to the Golgi apparatus and undergoes stepwise cleavage by S1P and S2P proteases to generate an active form that mediates transcriptional induction of *SESN2*. ATF-4: Activating Transcription Factor 4; ATF-6: Activating Transcription Factor 6; IRE-1 α: Inositol-Requiring Enzyme 1 α; JNK: c-Jun N-terminal Kinase; PERK: Protein kinase RNA-like Endoplasmic Reticulum Kinase; RIDD: Regulated IRE1α-Dependent Decay; XBP-1s: spliced X-box binding protein 1.

The endoplasmic reticulum (ER) is an important place for the post-translational modifications and proper folding of the transmembrane and secretory proteins, lipid biosynthesis, and calcium homeostasis. The folding capacity of the ER is closely monitored by three transmembrane proteins in the ER membrane inositol-requiring enzyme (IRE)-1α, protein kinase RNA-like endoplasmic reticulum kinase (PERK), and activating transcription factor 6 (ATF-6). When the ER is overwhelmed with unfolded or misfolded proteins due to physiological or pathological changes, these proteins signal unfolded protein response (UPR) (Figure 4). The UPR on one hand temporarily holds the translation of proteins to decrease the load on the already stressed ER, and on the other hand, it tries to increase the capacity of folding by signaling increased expression of components of the ER folding machinery, including chaperones. The UPR also increases the expression of stress-fighting molecules to maintain cellular homeostasis. If the stress response in the ER is beyond control, the UPR also serves as a safety valve to initiate apoptosis of the cell. As SESN2 is one of the stress-inducible proteins, there is a growing interest in studying the regulation of SESN2 by the UPR. Jegal et al. showed that the ER stress, induced by tunicamycin, can increase the mRNA and protein expression, as well as *SESN2* promoter-driven luciferase activity in a hepatocyte cell line HepG2. Among the canonical transcription factors of the UPR, ATF-6α was the most potent in the transcriptional activation of *SESN2*. The ectopic expression of ATF-6α increased the reporter gene activity while knockdown or silencing of *ATF-6α* blunted the SESN2 induction in response to tunicamycin. The induction of SESN2 protected the HepG2 cells from ER stress [40]. Although chemical inhibition of IRE-1α or PERK did not show any effect on SESN2 induction in this study, other studies also reported a role for these two ER effectors in the induction of SESN2 [41,42,43].

Park et al. previously showed that the chronic ER stress on the HepG2 cells in vitro and on mouse livers in vivo can increase the expression of SESN2 by CCAAT/enhancer-binding protein beta (CEBPB), a transcription factor downstream of PERK. The increased expression of SESN2 reduces ER stress and prevents mice from steatohepatitis and liver damage due to high-fat diet-induced (HFD) obesity. The other transcription factors downstream of PERK are ATF-4 and C/EBP homologous protein (CHOP). The expression of SESN2 was found to be associated with the expression of ATF-4 and CHOP in cancer cells undergoing treatment with Nelfinavir, an ER stress-inducing chemotherapeutic agent [42]. The ectopic expression of ATF-4 upregulated SESN2, indicating that ATF-4 is an upstream regulator of SESN2. Triple-negative breast cancer cells with ATF-4 depletion also exhibited an attenuated SESN2 induction in response to peptidyl arginine deiminases inhibitors [43]. Saveljeva et al. also reported that the expression of SESN2 in response to ER stress is independent of p53 and depends on PERK and IRE-1α/XBP-1 arms of the UPR. While the pharmacological inhibition of PERK or IRE-1α, in combination or alone, reduced the expression of SESN2, the knockdown of *ATF-6* did not affect the increased expression of SESN2 in response to ER stress induced by thapsigargin [44]. Consistent with these findings the knockdown of *PERK* and *XBP-1* in mouse embryonic fibroblasts reduced the expression of SESN2 and made cells sensitive to death by ER stress.

From the reviewed work, it seems that the three arms of the UPR i.e., PERK, IRE-1α, and ATF-6, contribute to the regulation of SESN2 in response to ER stress. However, the most effective arm may depend on the type of ER stress-inducing agent, the duration of ER stress (acute or chronic), and/or the type of cell used to model the pathology.

### 3.4. Oxidative Stress

**Figure 5:**
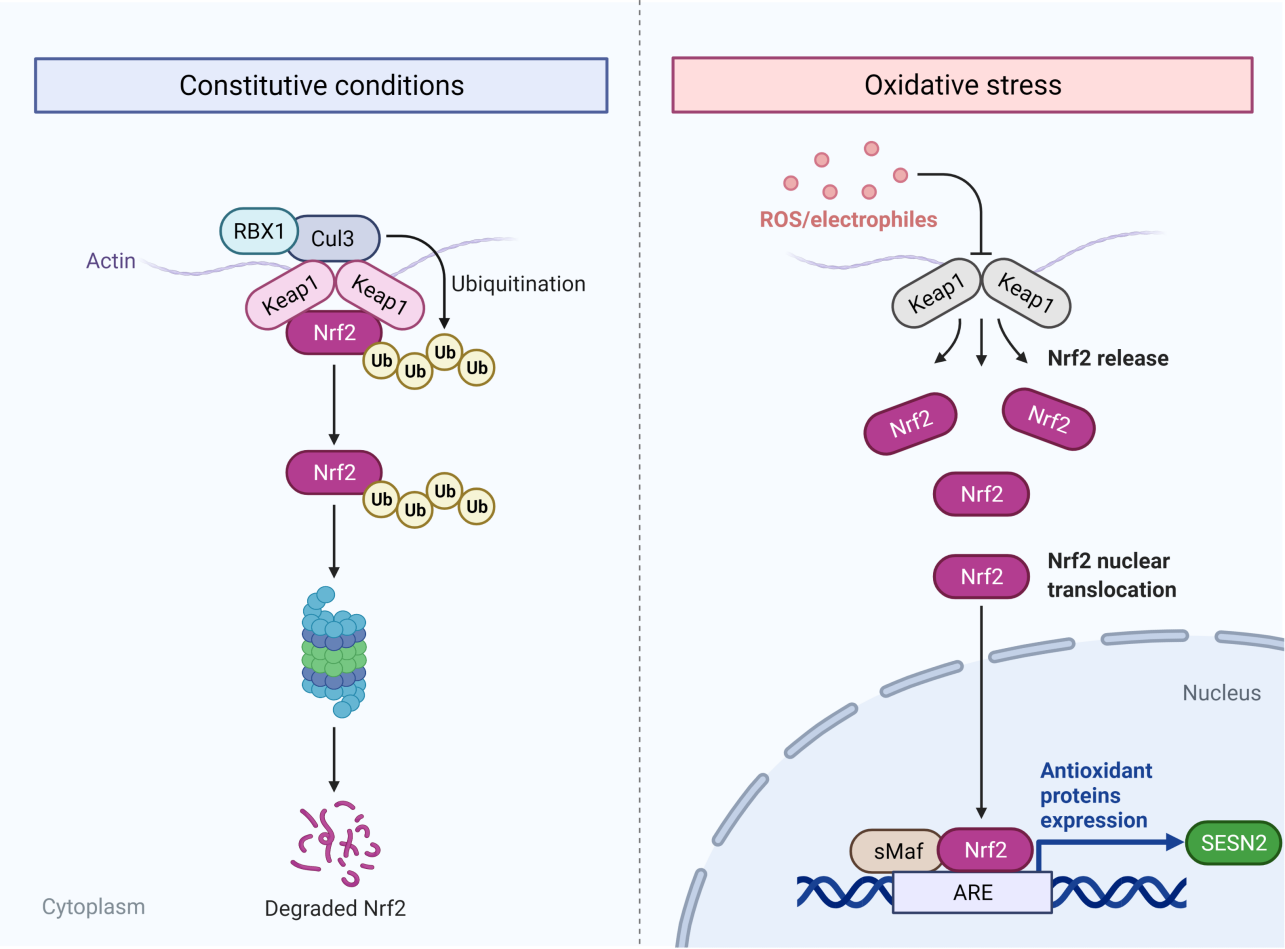
The regulation of SESN2 in response to oxidative stress. During constitute conditions, low levels of NRF2 are maintained due to ubiquitination mediated by KEAP-1 and proteasomal degradation of NRF2. ROS and electrophiles disrupt the binding of KEAP-1 with NRF2. NRF2 translocates to the nucleus and increases the expression of SESN2 by transactivation.

Oxidative stress, defined as an imbalance between oxidation and anti-oxidation systems, in addition to damaging nucleic acids can also damage other macromolecules, such as proteins, and lipids and, thus, greatly affect the aging and life span of the organism [45]. Oxidative stress is another trigger for the induction of SESN2. The observations from the initial experiments of Budanov et al. showed that the induction of SESN2 in response to hydrogen peroxide is independent of p53 because the presence of functional p53 is not required. Later, researchers showed that the induction of SESN2 in response to oxidative stress is dependent on the activation of NRF2. Chemical activators of NRF2 increase the expression of SESN2 only in the presence of a working NRF2 [46]. NRF2 is a transcription factor that mediates the antioxidant effects during stress by increasing the expression of antioxidant genes. Under normal conditions of oxidative balance, NRF2 remains bound to its negative repressor, Kelch-like ECH-associated protein 1 (KEAP-1), within the cytoplasm. The binding of the KEAP-1 homodimer with NRF2 facilitates proteolysis through its ubiquitination [47]. Oxidative stress and electrophiles cause the unbinding of the KEAP-1 from NRF2. The latter translocates to the nucleus for interaction with antioxidant response elements (ARE) for the transactivation of target genes (Figure 5). The *SESN2* promoter region contains ARE and NRF2 binds to this response element to increase the expression of SESN2 [46]. SESN2 is also shown to positively regulate the NRF2 signaling by P62-dependent autophagosomal degradation of KEAP-1 which sets NRF2 free for its nuclear translocation [10]. In turn, NRF2 increases the expression of SESN2 in a positive feedback manner [46].

## 4. Downstream Pathways Affected by SESN2

The downstream effects of SESN2 induction are mediated either by the intrinsic antioxidant activity of SESN2 or by increasing AMPK and NRF2 activation and mTORC1 inhibition. SESN2 also signals autophagy as a downstream effect. A review of the downstream effector pathways of SESN2 is presented here.

### 4.1. Intrinsic Antioxidant Enzyme Activity of SESN2

**Figure 6:**
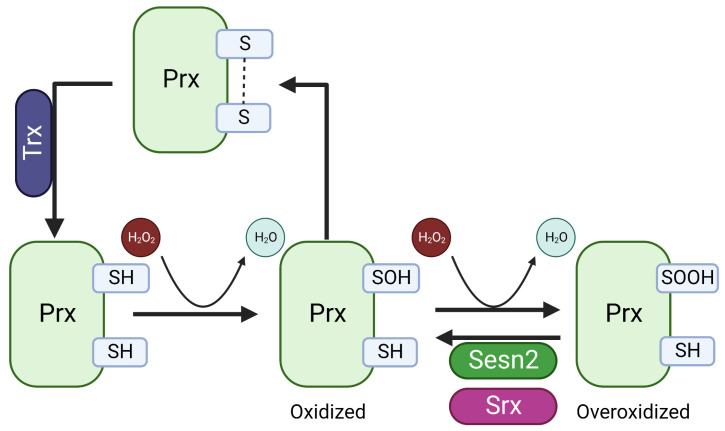
The generation of overoxidized peroxiredoxin by hydrogen peroxide and the reduction by SESN2 and sulfiredoxin. Oxidized Prx is reduced by Trx during low oxidative stress. High oxidative stress over-oxidizes Prx which is regenerated either by SESN2 or Srx. SESN2 is known to increase Srx expression through KEAP1/NRF2 pathway. Prx: Peroxiredoxin; Trx: Thioredoxin; Srx: Sulfiredoxin; SH: Reduced cysteine; SOH: Oxidized cysteine; SOOH: Overoxidized cysteine.

The NTD domain of SESN2 contains five alpha-helical conserved regions with sequence similarity to the C-terminal alpha-helical domains of AhpD [19]. AhpD is a prokaryotic protein with organic alkyl hydroperoxide reductase activity and is also required for the regeneration of AhPC, a peroxiredoxin family protein (Prx) oxidized during the reduction of peroxides and reactive nitrogen species (Figure 6) [48,49]. Based on sequence and structural similarity and the role of SESN2 as an antioxidant, it was natural to think of SESN2 as functionally similar to AhpD. Unlike AphD, which contains two cysteine residues required for the regeneration of AhPC, SESN2 contains only one proximal Cys125 and the mutation of this cysteine residue to serine abolished the activity of SESN2 to protect from oxidative stress [19]. In eukaryotes, the Prx pathway has evolutionary benefits to allow the functioning of peroxide as a signaling molecule. In eukaryotes, during the correction of the basal level of oxidants, the peroxidatic cysteine of Prx is oxidized to Cys-SOH which then forms a disulfide bridge with the resolving cysteine of the other dimer. Thioredoxin (Trx) reduces this disulfide bridge to reproduce Prx. However, in cases of high oxidative stress, the Cys-SOH form of Prx can over-oxidize to Cys-SO2H. Although the initial experiments by Budanov et al. showed the reductase activity of SESN2 on Prx, later studies could not confirm this observation [50]. The alkyl hydroperoxide reductase activity of SESN2 was confirmed by the reduction of cumene hydroperoxide, but cumene hydroperoxide is not an endogenous peroxide [51]. It is now well-agreed that SESN2 is not the stand-alone reductase for Prx but helps in the reduction of the overoxidized form of Prx indirectly by increasing the expression of sulfiredoxin (Srx) and other antioxidant mechanisms [50].

### 4.2. Inhibition of mTORC1

SESN2 affects the mTORC1 pathway in two different ways [8,20,30,31]. SESN2 facilitates the activation of AMPK which, in turn, activates kinase for tuberous sclerosis 2 protein (TSC2) and thus prevents the activation of mTORC1. SESN2 is shown to directly interact with GATOR2 and free GATOR1 from the inhibitory control of GATOR2. GATOR1, in turn, keeps mTORC1 inactive. Both pathways are reviewed here separately.

#### 4.2.1. SESN2-AMPK-TSC2-RHEB-mTORC1 Axis

The induction of SESN2 in response to different stimuli provides defense against the initiating stress by modulating the activity of two important downstream targets, mTORC1 and NRF2. Budanov and Karin showed that SESN2 connects p53 to mTORC1 in response to DNA-damaging stress. SESN2 activates AMPK by its phosphorylation at the Thr172 residue. TSC2 is a GTPase activating protein (GAP) for the mTORC1 regulatory protein RAS homolog enriched in the brain (RHEB). The activation of the GTPase activity of RHEB by TSC2 hydrolyzes RHEB-bound GTP to GDP and, thus, inactivates RHEB preventing, therefore, the activation of mTORC1. The inhibition of mTORC1 activation prevents the cell from the stress that results from the synthetic activity necessary for growth and proliferation of the cell. Even in the absence of TSC2, cells remain sensitive to AMPK. This is because AMPK can directly phosphorylate two well-conserved serine residues of the regulatory associated protein of mTOR complex 1 (RAPTOR) and, thus, inhibits mTORC1 [52]. The precise mechanism of AMPK’s activation by SESN2 was revealed later when immunoprecipitation studies showed the association of SESN2, AMPK, and LKB1, an upstream regulator of AMPK during ischemia. SESN2 worked as a scaffolding protein to increase the interaction between LKB1 and AMPK [6].

#### 4.2.2. SESN2–GATOR2–GATOR1–RRAG–mTORC1 Axis

Almost six years later, three independent research groups showed that the SESN2-AMPK-TSC2-RHEB-mTORC1 axis is not the only pathway for the control of SESN2 over mTORC1. SESN2 can also regulate the activity of mTORC1 through another GTPase belonging to the RAAG family which exists as an RRAG-A/B or RAAG-C/D heterodimer. The activation of RRAGs is dependent on the binding of GTP to RRAG-A/B and GDP to RRAG-B/C. The active heterodimers can bind with mTORC1 directly by RAPTOR and upon appropriate stimulation favoring cell growth and proliferation, stimulates the translocation of mTORC1 from the cytoplasm to the lysosomal membrane. RHEB then can activate mTORC1 at the lysosomal membrane.

The binding of GTP to RRAG-A/B and the lysosomal translocation of the mTORC1 is increased by the guanine nucleotide exchange factor (GEF) activity of the regulator complex [53]. Conversely, the GAP activates the GTPase activity of the RRAG-A/B, and guanidine nucleotide dissociation inhibitors (GDIs) bind to the GDP-bound state of the complex to prevent the exchange of GDP to GTP, thus keeping the complex in an inactive form. GATOR1 is a GAP for RAAG-A/B and itself is under the inhibitory control of GATOR2 in the super complex of GATOR. Parmigiani et al. [21] and Chantranupong et al. [54] showed that SESN2 can interact with GATOR2, thus, removing the inhibitory control of GATOR2 over GATOR1. Peng et al. [55] showed a different mechanism of RRAG-A/B inactivation and reported that SESN2 is a GDI for RAG GTPases. The interaction between SESN2 and GATOR2 is sensitive to leucine levels. In case of leucine sufficiency, SESN2 can bind with leucine, and the interaction between SESN2 and GATOR2 is disrupted, leaving GATOR2 to inhibit GATOR1, activating the RRAG-A/B and mTORC1 complex. Thus, SESN2 is a leucine sensor for the mTORC1 pathway [31].

The possibility that the inhibition of mTORC1 through the inhibition of RHEB by TSC2 and RAAG-A/B by GATOR1 are closely related cannot be ruled out. It has been shown previously that the GDP-bound inactive form of RRAG-A/B interacts with the TSC complex and brings the complex to the lysosomal membrane to interact with RHEB [56,57]. Thus, it is possible that inactive RRAG-A/B plays a role in the inactivation of RHEB as well and integrates the SESN2 signals from the AMPK–TSC2–RHEB and GATOR2–GATOR1–RRAG-A/B axes.

### 4.3. Activation of mTORC2

The interplay between SESN2 and mTORC2 is less studied as compared to mTORC1. By using a *SESN2* knock-down mouse model, Lee et al. showed that the presence of SESN2 is required for the activation of AKT in response to insulin. In carefully designed experiments, they showed that the activation of AKT by SESN2 is dependent on the presence of RPTOR independent companion of mTOR complex 2 (RICTOR), a core component of mTORC2. The interaction of SESN2 and GATOR2 was not only important for the inhibition of mTORC1 but was also required for the increase in the catalytic activity of mTORC2. The deletion or silencing of the GATOR2 domains which interact with either SESN2 or mTORC2 blunted the activation of AKT [10]. SESN2 was also found to bind to the pleckstrin homology (Ph) domain of AKT, favoring the recruitment of AKT to the plasma membrane [23]. In our own experience, we have observed a blunted activation of AKT by silencing *SESN2* in endothelial cells [58].

**Figure 7:**
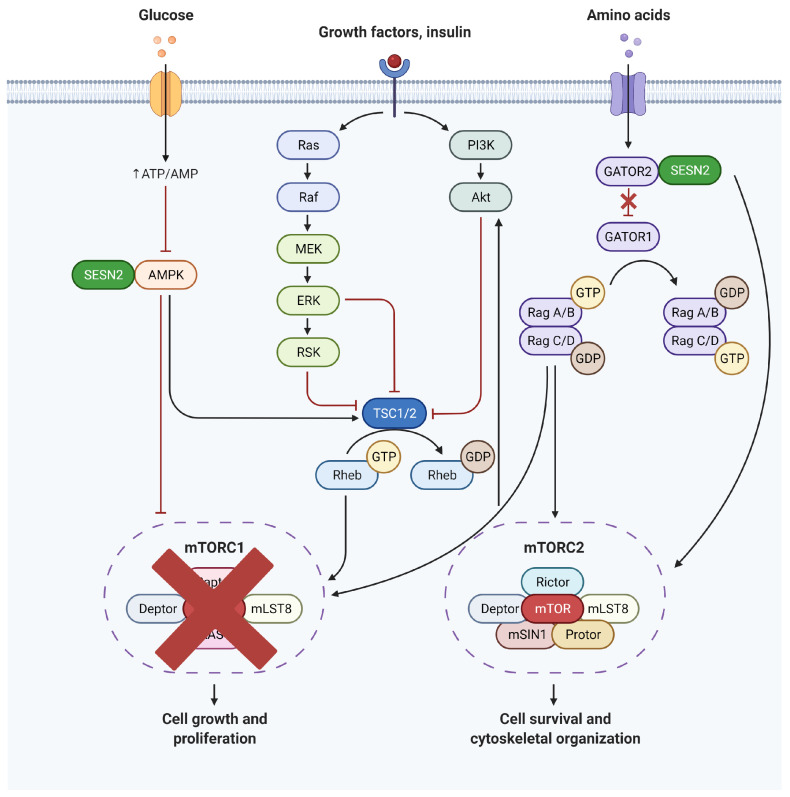
The Regulation of mTORC1, mTORC2, and AMPK by SESN2. The inhibition of mTORC1 by SESN2 is mediated either by AMPK or GATOR2. AMPK is activated by SESN2 which in turn directly inhibits mTORC1 or activates TSC2, a GAP for the mTORC1 regulatory protein Rheb. SESN2 is also known to inhibit GATOR2 in the absence of leucine so that GATOR1 can activate the GTPase activity of RAAG and inhibit mTORC1. SESN2-GATOR2 complex also interacts with RICTOR to increase the catalytic activity of mTORC2 and activate AKT signaling. Akt: Ak strain transforming; AMPK: AMP-activated Protein Kinase; ERK: Extracellular signal-Regulated Kinase; GATOR1: Gap Activity TOward Rags 1; GATOR2: Gap Activity TOward Rags 2; GDP: Guanosine DiPhosphate; GTP: Guanosine TriPhosphate; MEK: Mitogen-activated protein kinase; PI3K: Phosphoinositide 3-kinase; Raf: Rapidly accelerated fibrosarcoma kinase; Rag: Ras-related GTP-binding protein; Ras: Rat sarcoma kinase; Rheb: Ras Homolog Enriched in Brain; RSK: 90 kDa Ribosomal S6 Kinase; SESN2: Sestrin2; TSC1/2: Tuberous Sclerosis Complex 1/2.

The inhibition of mTORC1 at one end and the activation of mTORC2 and AKT at the other shows that SESN2 integrates pro-survival signals from the AKT pathway while keeping the mTORC1 on a short leash to prevent the overdue stress that accumulates due to translational activity within the cell. A schematic representation of the effects of SESN2 on mTORC1 and mTORC2 is presented in Figure 7.

### 4.4. KEAP-1-NRF2 Axis

The antioxidant properties of SESN2 were initially credited to the peroxiredoxin reductase activity of SESN2 [19], but later studies could not confirm this finding [50]. However, SESN2 increases the expression of sulfiredoxin, a reductase for hyperoxidized peroxiredoxin under the transcription control of NRF2 [22,59]. Bae et al. studied the missing link between SESN2 and NRF2 and showed that the degradation of KEAP-1 by autophagy is promoted by SESN2. KEAP-1 is a negative regulator of NRF2 in the cytoplasm and acts as a substrate adapter for the ubiquitination of NRF2 by the ubiquitin ligase complex. The proteasomal degradation keeps NRF2 levels low in case of non-stress states. Oxidants and electrophiles disrupt the correct conformational binding between NRF2 and KEAP-1 necessary for the ubiquitination and degradation of NRF2 and facilitate nuclear translocation and induction of transcription of antioxidant genes (Figure 5). The induction of KEAP-1 degradation by forced induction of SESN2 is dependent on P62 [22]. P62 or sequestosome 1 (SQSTM1) is an autophagy adapter with a KIR domain capable of binding with and tagging KEAP-1 for autophagic degradation. KIR domain resembles the ETGF motif of NRF2 and thus P62 competes with NRF2 for binding with KEAP-1. The interaction between KEAP-1 and P62 is not strong enough for the degradation of KEAP-1 with the autophagy substrate unless the concentration of KEAP-1 is enough to drive the equilibrium in the bound state. Thus, SESN2 appears to be a scaffolding protein to strengthen the interaction between KEAP-1 and P62.

### 4.5. SESN2 and Autophagy

Autophagy is a protective mechanism by which the cells degrade damaged cytoplasmic organelles, proteins, and lipids to protect cells from stress and maladaptive cellular signals, as well as allow the generation of energy, especially during nutrient depletion [7]. In this process, the cytoplasmic content is encapsulated within the autophagosomes, double-membraned vesicles, which then fuse with lysosomes for degradation of their cargo content. Mitophagy is the autophagic elimination of malfunctioning mitochondria protecting the cell against aberrant ROS production. Autophagy-activating kinase (ULK1) is an important kinase in the formation of autophagosomes and regulates the formation of autophagophores [54]. SESN2 is a positive regulator of autophagy through the activation of AMPK and the inhibition of mTORC1 [24]. AMPK phosphorylates Ser317 and Ser777 of ULK1 activating it and inducing autophagy [60]. In contrast to AMPK, mTORC1 has an important role in negatively regulating autophagy as it inhibits ULK1 by its phosphorylation at Ser757 and disrupts the interaction between AMPK and ULK1 [60]. SESN2 also facilitates the phosphorylation of P62 by ULK1 and acts as a scaffolding protein [61]. P62 is an autophagy substrate and phosphorylation of P62 increases its binding affinity to targets, including KEAP-1, increasing their autophagic degradation [61].

## 5. SESN2 and Cardiovascular Diseases

### 5.1. Hypertension

Hypertension, dyslipidemia, and diabetes are among the leading and preventable risk factors for cardiovascular diseases. Essential hypertension is the most common type of hypertension and accounts for more than 95% of hypertension cases. The increase in oxidative stress and inflammation with growing age leads to endothelial dysfunction, making pressure-regulating vessels unresponsive to dilating stimuli. Moreover, the blood pressure regulatory function of the kidneys is disrupted by increased ROS and is associated with the chronic activation of the adrenergic nervous system and the renin–angiotensin–aldosterone system (RAAS), and increased reabsorption of sodium and water from the renal tubules, leading to volume and pressure overload. SESN2 has been shown to mediate the inhibitory effects of dopamine D2 receptors on ROS accumulation. The silencing of *SESN2* in the mouse kidney increased ROS production and blood pressure in *SESN2*-deficient mice [62]. Angiotensin-II (Ang-II) is an effector of RAAS that not only increases blood pressure but also increases oxidative stress in the endothelial cells, making them hypertrophic and contributing thus to endothelial dysfunction. SESN2 has been shown to prevent the deleterious effects of Ang-II on the endothelium [63]. Ang-II increased the expression of SESN2 in human umbilical vein endothelial cells (HUVEC) in a time- and dose-dependent manner. Knockdown of *SESN2* by siRNA increased oxidative stress and reduced the viability of endothelial cells in response to Ang-II. The increase in SESN2 was dependent on JNK/c-Jun pathway as overexpression of c-Jun increased the luciferase activity under the *SESN2* promoter [63]. Increased circulating levels of SESN2 have been reported in hypertension patients [64]. The compensatory increase in SESN2 expression in response to Ang-II may serve as a protective mechanism to keep blood pressure within physiological limits.

### 5.2. Atherosclerosis

Atherosclerotic plaque formation is a complex process with the involvement of endothelial cells, vascular smooth muscle cells (VSMC), and the immune system. Hyperglycemia, dyslipidemia, hypertension, immune dysregulation, and endothelial dysfunction have been implicated in the development and progression of atherosclerotic plaques. SESN2 protects against the development and progression of plaque formation and higher levels of SESN2 plasma levels have been found in patients with carotid plaques and are associated with the severity of carotid stenosis [65]. Endothelial dysfunction is a well-established response to cardiovascular risk factors, such as hypertension, hyperglycemia, and dyslipidemia, and precedes the development of atherosclerosis. SESN2 has been shown to protect against endothelial dysfunction caused by different types of cellular stresses. Fatima et al. showed that *SESN2* silencing can aggravate the effects of ER stress induced by thapsigargin. Silencing of *SESN2* increased oxidative stress, reduced viability of endothelial cells, and dysregulated NRF2, AMPK, and mTORC1 signaling pathways [58].

Macrophages play an important role in the formation and development of atherosclerotic plaques. Macrophages are derived from circulating monocytes and the expression of adhesion molecules on endothelial cells facilitates the capture, activation, transport, and polarization of these monocytes into the subendothelial tissue to become tissue macrophages. SESN2 has been shown to reduce the expression of adhesion molecules on the surface of HUVECs, and monocyte (THP-1) activation and polarization in vitro [66,67]. Knockdown of *SESN2* increased ROS, ER stress, and the secretion of proinflammatory cytokines from endothelial cells and monocytes in response to lipopolysaccharides (LPS) exposure [66]. The expression of adhesion molecules on the endothelial cells and the adhesion of monocytes to endothelial cells were increased. At the molecular level, the decreased activation of AMPK and increased phosphorylation of nuclear factor kappa B (NFκB) was observed. The activation of AMPK by AICAR (5-aminoimidazole-4-carboxamide-1-β-D-ribofuranoside) abrogated the effects of *SESN2* silencing and LPS treatment on these cells. Similar results were obtained from aorta samples of mice. Hyperglycemia and dyslipidemia increase the risk of atherogenesis and SESN2 has been shown to decrease the activation of monocytes (THP-1) and the expression of pro-inflammatory markers in these states [67]. Hyperglycemic or dyslipidemia conditions increased the adhesion of monocytes to endothelial cells, the polarization of monocytes to M1 macrophages, and the formation of foam cells. The silencing of *SESN2* and AMPK inhibition by compound C worsened the situation by increasing the phosphorylation of mTOR. The accumulation of lipids and the formation of foam cells were increased by the inhibition of autophagy. Hu et al. observed an increased expression of SESN2 in the macrophage cell line Raw264.7 upon exposure to oxidized low-density lipoproteins (OxLDL) in a time- and dose-dependent manner. The silencing of *SESN2* by siRNA increased ROS production and apoptosis of the cells. Death of macrophages is characteristic of advanced plaques and contributes to the formation of necrotic core and destabilization of the plaque. Mechanistically, an increase in SESN2 expression in response to OxLDL was dependent on JNK/c-Jun pathway, as inhibiting the activity of the pathway abolished the effects of OxLDL on the expression of SESN2 [68].

Precise control of the proliferation and apoptosis of VSMCs is required for healthy vessels. The excessive proliferation of VSMCs can lead to atherosclerosis while their apoptosis may cause destabilization and rupture of atherosclerotic plaque. The induction of SESN2 by melatonin has been shown to reduce the proliferation of VSMCs by inhibiting mTORC1 and reducing ROS [69]. Melatonin increased the expression of SESN2 through C/EBPβ by inducing mitochondrial energetic stress.

### 5.3. Ischemia/Reperfusion Injury

The increased oxygen demand of the myocardium or limited supply due to plaque build-up in the coronary arteries leads to myocardial ischemia which may progress to infarction if reperfusion is not achieved. Reperfusion should, in principle, restore the dysfunction of the myocardium, but paradoxical damage to the tissue after the restoration of the blood flow is a well-known phenomenon, known as ischemia-reperfusion (I/R) injury [70]. Generation of oxygen-free radicals upon the resupply of oxygen, endothelial dysfunction and microvascular injury, alterations in calcium handling, and myocardial metabolism are mediators of I/R injury. There is increasing evidence that SESN2 is important in preventing the heart from ischemia/reperfusion injury. Higher SESN2 levels and increased oxidative stress were observed in response to I/R as compared to the normoxic hearts [71]. Knockdown of *SESN2* worsened oxidative stress and increased the infarct size and cardiac dysfunction in I/R. The re-introduction of *SESN2* in the knockout hearts by the adenoviral expression system rescued the hearts from I/R injury by increasing the expression of SESN2 and reducing oxidative stress. During ischemia, SESN2 protein expression was found to be increased in the adult cardiomyocytes just after 5 minutes, indicating decreased degradation of the protein instead of increased de novo synthesis [6]. In *SESN2*-deficient mouse hearts, I/R greatly enhanced the infarct size. Ischemic AMPK activation was found to be impaired in the *SESN2*-deficient hearts. Mechanistically, SESN2 served as a scaffolding protein to facilitate the interaction of AMPK with its upstream kinase LKB1. The cardioprotective effects of AMPK during and after ischemia are well-established and ischemia-induced AMPK activation decreases with age which makes the aging hearts more susceptible to ischemic injury. Quan et al. studied the role of SESN2 in decreased activation of AMPK and showed changes in downstream substrate metabolism in response to ischemia. The expression of SESN2 decreased with age in mouse hearts and corresponded with the decreased activation of AMPK in response to ischemia [11,12]. The uptake of glucose and the rate of glucose oxidation was significantly impaired in the aged wild-type hearts and young *SESN2*-deficient hearts. The phenotype observed in *SESN2*-deficient hearts was similar to wild-type aged hearts. An aging-like phenotype of mouse hearts in response to *SESN2* deficiency was also reported in other more recent studies [72,73]. Transcriptomic changes in the *SESN2*-deficient hearts were also like aged wild-type hearts when challenged with I/R indicating an age-related decline in SESN2 levels and a decreased tolerance of the myocardium to I/R. *SESN2* deficiency in the hearts increased oxidative stress, provoked an immune response, and resulted in structural changes similar to those observed in aged hearts under physiological conditions [73].

The overexpression of SESN2 in the hearts with the coronary delivery of an overexpression plasmid DNA improved the tolerance of the hearts to I/R via enhanced activation of AMPK, improved uptake of glucose by increasing the translocation of GLUT4 to the plasma membrane and increased rate of glucose oxidation [11]. Regional myocardial ischemia in the hearts of aged mice and *SESN2*-deficient young mice decreased the activation of AMPK and downregulated PGC-1α [12]. PGC-1α is a mediator of mitochondrial biogenesis. The downstream effectors of PGC-1α, TFAM, and UCP2, were impaired and apoptotic flux markers, AIF and Bax/Bcl-2, were upregulated in the aged and *SESN2*-deficient hearts making them more susceptible to ischemic injury. A similar protective effect of SESN2 via the AMPK/ PGC-1α pathway has also been described in the cerebral I/R [74]. *SESN2* silencing by *SESN2*-specific siRNA duplexes exacerbated neuronal damage and increased infarct volume in response to ischemia modeled by the occlusion and reperfusion of the middle cerebral artery. A significant increase in oxidative stress with a corresponding decrease in mitochondrial biogenesis was also observed. The antioxidant effects of SESN2 on the heart in response to I/R were also attributed to a significant reduction in the activation of p38 mitogen-activated protein kinase (MAPK), extracellular signal-regulated kinase (ERK), and JNK which was observed following the overexpression of SESN2 in isolated cardiomyocytes [71].

In patients with coronary artery disease (CAD), plasma levels of SESN2 were found to be high and associated with the severity of CAD [75,76]. Higher circulating levels of SESN2 were also observed in diabetic patients [77]. However, diabetic patients with concurrent CAD had lower serum SESN2 as compared to diabetic patients without CAD.

### 5.4. Cardiac Hypertrophy and Heart Failure

Cardiac hypertrophy is an adaptive response to pressure or volume stress and can lead to heart failure. Increasing evidence suggests that SESN2 prevents hypertrophy and cardiac remodeling. Overexpression of SESN2 salvaged the neonatal heart cardiomyocytes from the hypertrophic effects of phenylephrine by decreasing the phosphorylation of MAP-kinases and mTOR [78]. Silencing of *SESN2* increased the hypertrophic marker, anti-natriuretic peptide (ANP), and cell surface area, indicating the protective role of SESN2 in phenylephrine-induced hypertrophy. The heart requires precise control of mTORC1. Hyperactivation of mTORC1 can increase the chance of postpartum cardiac hypertrophy [79]. As a regulator of mTORC1 activity, SESN2 is necessary to keep mTORC1 levels within control to prevent hypertrophy after birth. An RNA-binding protein (RBP), Zinc finger protein 36 like 2 (ZFP36L2) was found to inhibit mTORC1 to prevent hypertrophy in a p53-dependent manner by increasing the decay of MDM2 mRNA and increasing the expression of SESN2 [79]. The stabilization of p53 by chemical stabilizers could be used as a therapeutic strategy to prevent the hyperactivity of mTORC1 [80]. Nutlin-3, a chemical that destabilizes the interaction between p53 and MDM2 increased SESN2 levels and baseline expression of ZFP36L2 to rescue the heart from hypertrophic changes [79].

SESN2 also showed a protective role against oxidative stress and atrial fibrosis in a cell model of atrial fibrillation [81]. Collagen volume fraction was increased in patients and a positive association between SESN2 concentrations and oxidative stress in atrial fibrillation patients was found [81]. Overexpression of SESN2 increased the survival of HL-1 cells and prevented fibrosis by decreasing the proliferation of fibroblasts in response to Ang-II [63].

Cardioprotective effects of pentamethyl quercetin (PMQ) by increasing the concentration of SESN2 in a transverse aorta constriction-induced pressure-overload cardiac-remodeling model in mice, and an isoproterenol-induced neonatal rat cardiomyocyte hypertrophy model, have been reported [82]. Similar protective effects of PMQ on cardiac remodeling were also observed in monosodium glutamate-induced obese mice through SESN2/KEAP-1/NRF2 pathway [83]. The increased expression of SESN2 by PMQ not only prevented cardiac remodeling but also improved metabolic disorders in the mice. Obese patients are at higher risk of developing diabetes and cardiovascular complications. In a recent study, diastolic function was studied in obese mice and cardiospecific *SESN2* knockout exacerbated the impairment of the diastolic function which was preserved by overexpression of SESN2 [26]. *SESN2* deletion increased fibrosis, cellular damage, inflammation, and ROS levels. Increased NRF2 and SESN2 expression were observed in the human cardiac tissues of obese individuals. Such an increase could be a compensatory response to an increased ROS. Empagliflozin has been shown to reduce hypertrophy, fibrosis, and cardiac dysfunction in a SESN2-dependent manner in an obese mouse model [84]. However, the dependency of Empagliflozin on SESN2 for the cardioprotective effects was partial, indicating the involvement of other pathways as well. In the fatty liver of mice, Empagliflozin has been shown to activate the AMPK/mTOR pathway and decrease lipid accumulation in hepatocytes in a SESN2 dependent manner [85].

A functioning ER is of particular importance for the contractile function of the cardiomyocytes as it maintains calcium homeostasis within the cell. ER stress can alter this homeostasis resulting in cardiac dysfunction which is at the core of many heart diseases including hypertrophy, ischemic heart disease, and heart failure. ER stress in the mouse heart induced by tunicamycin increased the myocardial volume and reduce the ejection fraction of the heart [25]. The defects in contractile function were due to alterations in calcium homeostasis as the decay rate of calcium intracellular was greatly reduced. The dysfunction of the heart was more pronounced in the *SESN2*-deficient mice. The genetic deletion of *SESN2* worsened the ER stress by decreasing the activation of AMPK, increasing the activation of mTORC1, and decreasing autophagy process [25]. Further studies are, however, required to examine the role of SESN2 overexpression on autophagy induction and protection from ER stress and cardiac hypertrophy.

Inflammation also plays an important role in inflammation-mediated cardiomyopathy and cardiac remodeling. The inflammatory condition induced by LPS in H9c2 cardiomyocytes required functional levels of SESN2 for AMPK activation, antioxidant gene *SOD2*, and catalase expression and protection against increased ROS levels [86]. MMP2 and MMP9 expression and cell death were also increased by *SESN2* knockdown. AICAR, an AMPK activator, prevented these disturbances. Consistent results were obtained in vivo in a mouse model where *SESN2* deletion increased the expression of cardiac fibrotic factors, collagen I and III, in addition to MMP2 and MMP9. High serum levels of SESN2 clearly could distinguish septic shock patients from healthy controls, whereas low circulating levels of SESN2 are related to cardiac dysfunction to some extent but are not an independent influence factor for septic cardiomyopathy [87]. Low circulating levels of SESN2 were shown to be useful in predicting clinical outcomes in patients with septic cardiomyopathy [88].

## 6. Conclusions

The ability of the body to respond to stress in an adequate and timely manner is important for the viability and functioning of the cells and to prevent the accumulation of cellular lesions. Cellular proteins and molecular pathways induced in response to stress either protect the cell from the damaging effects or help the cell to die in peace to prevent chronic inflammation. The response of these inducible proteins to stress is known to decline with age, but other epigenetic and genetic factors cannot be ruled out. The declined response to stress results in age-related pathologies, such as CVD, and modulating the expression or activity of stress-inducible proteins presents an interesting opportunity to prevent or treat these pathologies. SESN2 is a stress-inducible protein that acts as a protective shield against CVD directly or by modulating the activity of different cellular pathways. The expression of SESN2 has been shown to increase by some natural products, e.g., resveratrol, eupatilin, luteolin, quercetin, and pentamethyl quercetin [82,89,90,91,92]. Empagliflozin also modulates the activity of downstream pathways in SESN2 dependent manner [84,85]. The modulation of the activity of SESN2 is possible by small molecules which bind in the leucine binding site of SESN2. NV-5138, a leucine analog for SESN2 which selectively activates mTORC1 in the brain is under development by Navitor Pharmaceuticals [93]. An antagonist of leucine effects on SESN2 in principle can inhibit mTORC1 even in the presence of leucine sufficiency. The structural basis for the interaction of SESN2 with other proteins, such as AMPK and LKB1, needs further research to find small molecules capable of stabilizing such interactions. Such molecules will open a novel avenue in the discovery of drugs against CVD and age-related pathologies.

## Data Availability

Not applicable.

## Abbreviations

The following abbreviations are used in this manuscript:

CTD	C-terminal domain
LD	Linker domain
NTD	N-terminal domain
HRE	Hypoxia Response Element
VHL	Von Hippel–Lindau tumor suppressor
MDM2	Mouse Double Minute homolog
Ub	Ubiquitin
ATF-4	Activating Transcription Factor 4
ATF-6	Activating Transcription Factor 6
IRE-1α	Inositol-Requiring Enzyme 1 α
JNK	c-Jun N-terminal Kinase
PERK	Protein kinase RNA-like Endoplasmic Reticulum Kinase
RIDD	Regulated IRE1α-Dependent Decay
XBP-1s	spliced X-box binding protein 1
Prx	Peroxiredoxin
Trx	Thioredoxin
Srx	Sulfiredoxin
SH	Reduced cysteine
SOH	Oxidized cysteine
SOOH	Overoxidized cysteine
Akt	Ak strain transforming
AMPK	AMP-activated Protein Kinase
ERK	Extracellular signal-Regulated Kinase
GATOR1	Gap Activity TOward Rags 1
GATOR2	Gap Activity TOward Rags 2
GDP	Guanosine DiPhosphate
GTP	Guanosine TriPhosphate
MEK	Mitogen-activated protein kinase
PI3K	Phosphoinositide 3-kinase
Raf	Rapidly accelerated fibrosarcoma kinase
Rag	Ras-related GTP-binding protein
Ras	Rat sarcoma kinase
Rheb	Ras Homolog Enriched in Brain
RSK	90 kDa Ribosomal S6 Kinase
SESN2	Sestrin2
TSC1/2	Tuberous Sclerosis Complex 1/2
